# Can clinical decision support systems be an asset in medical education? An experimental approach

**DOI:** 10.1186/s12909-023-04568-8

**Published:** 2023-08-11

**Authors:** Sean D. Kafke, Adelheid Kuhlmey, Johanna Schuster, Stefan Blüher, Constanze Czimmeck, Jan C. Zoellick, Pascal Grosse

**Affiliations:** Charité – Universitätsmedizin Berlin, corporate member of Freie Universität Berlin and Humboldt-Universität zu Berlin, Berlin, Germany

**Keywords:** Medical students, Diagnosis, Medical decision-making, Clinical decision support systems

## Abstract

**Background:**

Diagnostic accuracy is one of the major cornerstones of appropriate and successful medical decision-making. Clinical decision support systems (CDSSs) have recently been used to facilitate physician’s diagnostic considerations. However, to date, little is known about the potential assets of CDSS for medical students in an educational setting. The purpose of our study was to explore the usefulness of CDSSs for medical students assessing their diagnostic performances and the influence of such software on students’ trust in their own diagnostic abilities.

**Methods:**

Based on paper cases students had to diagnose two different patients using a CDSS and conventional methods such as e.g. textbooks, respectively. Both patients had a common disease, in one setting the clinical presentation was a typical one (tonsillitis), in the other setting (pulmonary embolism), however, the patient presented atypically. We used a 2x2x2 between- and within-subjects cluster-randomised controlled trial to assess the diagnostic accuracy in medical students, also by changing the order of the used resources (CDSS first or second).

**Results:**

Medical students in their 4^th^ and 5^th^ year performed equally well using conventional methods or the CDSS across the two cases (*t*(164) = 1,30; *p* = 0.197). Diagnostic accuracy and trust in the correct diagnosis were higher in the typical presentation condition than in the atypical presentation condition (*t*(85) = 19.97; *p* < .0001 and *t*(150) = 7.67; *p* < .0001).These results refute our main hypothesis that students diagnose more accurately when using conventional methods compared to the CDSS.

**Conclusions:**

Medical students in their 4^th^ and 5^th^ year performed equally well in diagnosing two cases of common diseases with typical or atypical clinical presentations using conventional methods or a CDSS. Students were proficient in diagnosing a common disease with a typical presentation but underestimated their own factual knowledge in this scenario. Also, students were aware of their own diagnostic limitations when presented with a challenging case with an atypical presentation for which the use of a CDSS seemingly provided no additional insights.

## Background

Diagnostic accuracy is one of the major cornerstones of appropriate and successful medical decision-making [1, 2]. Already students of medicine are gradually getting familiarised with the crucial skill of making their own diagnosis [3, 4] and to critically appraise them [5, 6]. Didactical approaches teaching students on how to arrive at diagnostic conclusions have certainly differed depending on factors such as time, country, and medical institution [7, 8]. In addition, technological innovations have also contributed to changing procedures in the teaching of how to make a proper diagnosis [9]. One such example for technological assistance would be digitised clinical decision support systems (CDSSs) which are now extensively used by clinicians [10] and patients [11] alike. The potential usefulness of CDSS in the teaching of diagnostic processes for medical students will be further explored in this study.

CDSSs are software which are meant to offer impersonal support in medical decision-making in order to facilitate diagnostic processes [12]. Prototypes of current CDSSs were already developed as early as in the 1950s [13, 14]. Over time these software have gradually become more refined, in particular with the advancement of digital technologies and by using artificial intelligence based on huge data samples [15, 16] instead of reductionist diagnostic algorithms alone. Today, the emergence of smartphones [17] have made CDSSs easily accessible for the general public in the form of symptom checkers [18]. Given the broad dissemination of such CDSSs their performance and the potential usefulness in medical practice in general has already been studied extensively [19, 20]. Also, Berner et. al. found an accuracy of 52% to 71% when comparing the performance of four CDSSs on over 100 challenging cases meaning these systems provided a right diagnosis out of a choice of predefined possible diagnoses [21]. Over time, CDSSs’ accuracy increased, as shown by Graber and Mathew who reported an accuracy of 98% measured through naming the right diagnosis in the first 30 possible diagnoses in over 50 different challenging cases [22]. But there seems to be a discrepancy when it comes to uncommon presentation of diseases. As an example, Hill et al. found that symptom checkers listed the right diagnosis first for common presentations in 42% in contrast to just 4% for uncommon presentations [23]. It is of note that the accuracy of symptom checkers seems to have stagnated over the last decade [24].

As digitised CDSSs seem to be of major relevance in the nearer future we have been wondering whether the use of CDSSs also were an asset for medical students when they develop their diagnostic decision-making skills and the process related to diagnostic decision-making. As such, CDSSs were to be viewed as an educational tool that offer medical students additional avenues towards medical decision-making [25]. However, to date, evidence has been scarce, if not contradictory whether these software will eventually fulfill such promises [26, 27].

To the best of our knowledge our study is the first one to explore the usefulness of CDSSs in medical education with regards to the influence of a CDSS on students’ diagnostic accuracy and their trust in their own diagnoses as outcomes of their decision-making process. Most studies using supporting devices for medical decision-making almost exclusively focus on rare diseases or challenging if not unusual cases [21]. In contrast, we opted for common diseases, one presenting with typical signs and symptoms, the other with an atypical presentation. Our aim was to appropriately contrast results and being able to compare them. As we intended to explore a potential usefulness of CDSSs in an educational setting, we needed diseases students are familiar with and to reflect the stage of their knowledge. We opted for the common and generally accepted approach of using clinical case vignettes to test students’ accuracy and trust [28, 29].

We expected students to solve the case with the typical presentation of a common disease with more diagnostic accuracy than the case with the atypical presentation, irrespective of whether they used conventional methods as defined above or a CDSS. Our primary hypothesis was that diagnoses using conventional methods were on average more accurate than diagnoses with a CDSS as students were still unfamiliar with the CDSSs and their approach to use this software appropriately. Further, we hypothesised that students would have greater trust in their own diagnostic accuracy when relying on CDSS as they lack the personal experience in medical decision-making in this early phase of their career as future doctors.

## Methods

The study was pre-registered with the Open Science Framework [30]. The Charité ethics committee approved of the study.

### Study setting

We studied medical students in their 4^th^ and 5^th^ year of their studies (semesters 7 to 10). Medical studies at our university last a total of six years with the last year being an internship year. Since we have an integrated curriculum there is no strict separation between preclinical or clinical studies. We only included more advanced students as the task to come to diagnostic conclusions would be far too challenging for students at the beginning of their studies. As such we created a relatively homogenous sample of students. Assessment was performed in the setting of peer-teaching tutorials which last for three hours. A broad variety of elective courses are offered to students from which they can actively choose according to their own interests. At the end of our course, students received four tutorial credit-points, of which they have to collect 60 during the first five years of their studies. Credit points have no effect on students’ grades. Each tutorial was limited to 20 participants to provide appropriate supervision and to ensure the participants’ active participation from April 2020 to November 2020. Students not fitting our inclusion criteria for the study were, however, not excluded from the tutorial itself. In all our classes the same two instructors, a student peer teacher in her 4^th^ year of their medical studies and an experienced instructor, held the tutorial in a digital format via Microsoft Teams, as regulations regarding the CoViD-19-pandemic did not allow a physical setting. Tutorials at our university are always held by student peer-teachers. In the case of our study we added an experienced instructor to explain all aspects related to the study in order to separate the factual teaching by the student peer-teacher from the technical details of the study. Students were asked to leave their cameras on to ensure a classroom setting as well as supervision. At the beginning of a class the instructors introduced the participating students to the use of the CDSS to ensure a working application. Once all participants had familiarised themselves with the CDSS, one of the paper cases was provided alongside with a questionnaire using the software Unipark^®^. We provided an information sheet for the students prior their participation and allowed time for questions. In that sheet, we informed students that their participation was voluntary and that prematurely ending the study would not create negative outcomes – i.e., students would still receive their credit points for the tutorial. We also informed students that their data would be collected without the possibility to identify individuals later, and that it can be published in anonymous form.

Thirty-four medical students (73% female) aged *M* = 25.29 years (*SD* = 4.15 years) in semester *M* = 8.31 (*SD* = 1.82) participated in a pilot run of the study. The potential usefulness of the CDSS in general was not discussed with the students prior to the study. The students were given 60 min within the tutorial to solve their respective case in both conditions. Case and order were randomly selected before each tutorial date. Participation in the study itself was voluntary and had no effect on receiving credit-points, as long as active participation during the rest of the course was provided. We did not provide final diagnoses during courses as to not skew results in later dates of the tutorial. After the pilot run, we adapted the questionnaire to include the case description repeatedly in the two within-subject conditions and specified some formulations in the instructions. Feedback after the pilot run was that 60 min were sufficient to solve the task at hand. The process itself remained the same for the main study.

### Study design

**Fig. 1:**
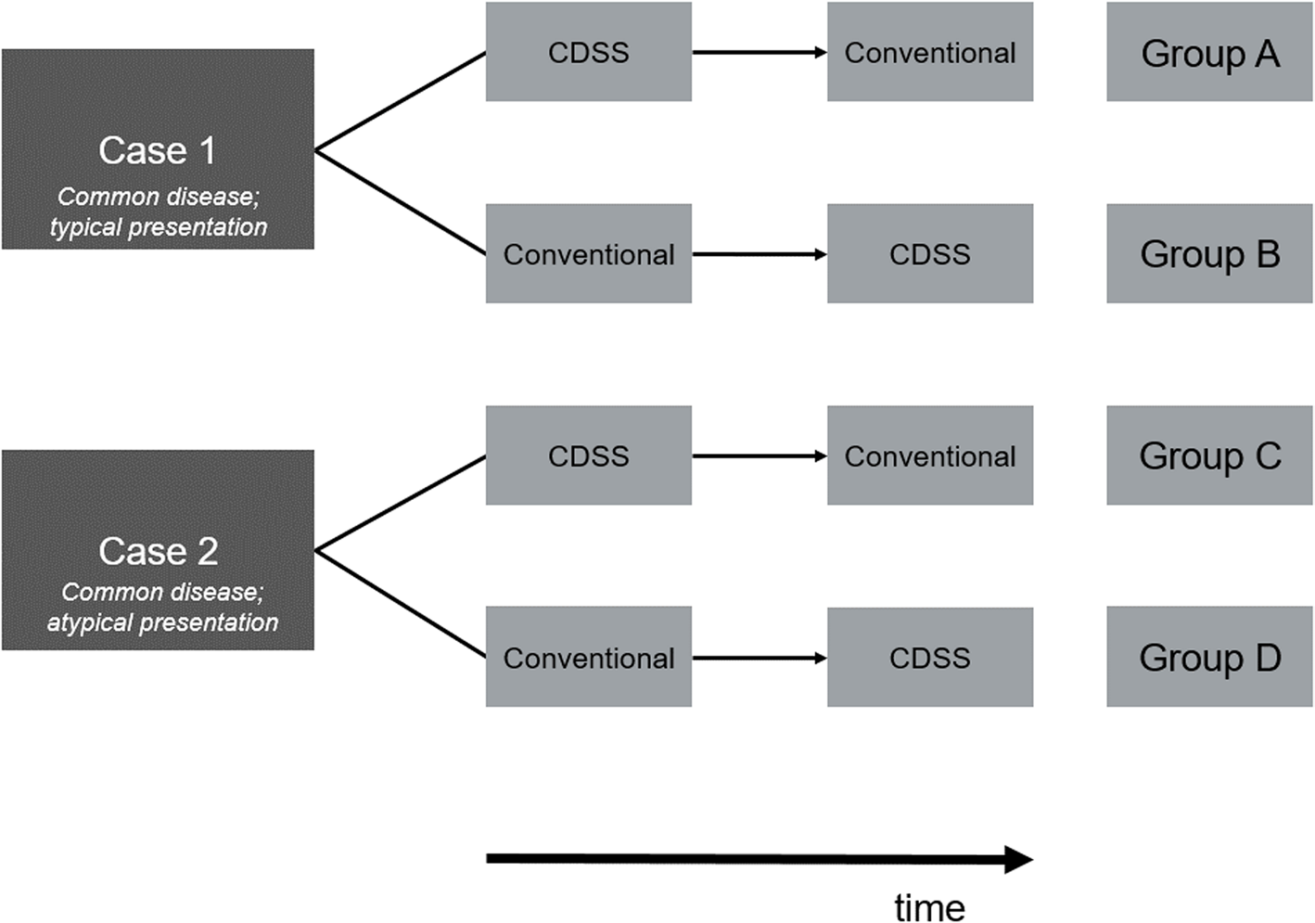
Design of the experiment and subsequent grouping. Groups A, B, C, and D present the four conditions based on the between factor case (typical presentation vs. atypical presentation) and the between factor order (CDSS first or conventional method first). Groups A and B solved case 1 in the opposite order of task and groups C and D solved case 2 correspondingly

The study was set up as a 2x2x2 between- and within-subjects cluster-randomised control trial. Participating students were clustered into groups, and these groups were randomly assigned to one of four conditions based on case and order. The two paper cases as between factors were a common disease in typical presentation (tonsillitis with throat ache and swollen red tonsils covered by a white exudate = case 1 [the case was adapted from a case-based learning sample from our university]) and a common disease in atypical presentation [31] (pulmonary embolism presenting with upper abdominal pain = case 2 [32]). In addition to the cardinal symptoms, in both cases a short medical history as well as the essential diagnostic findings at time of consultation were presented. Each participant had to solve his or her respective case twice – once with the help of a CDSS and once using conventional methods (within factor). Students were completely ignorant of the degree of difficulty and whether the paper case patients presented with typical constellation or not. Conventional methods were specified as to which may be used (textbooks, research literature, internet research, and similar resources), but not explicitly detailed in terms of e.g., order in which to use these materials allowing students to use any approach they were familiar with in the setting of a tutorial to solve the task. Conventional methods explicitly excluded the use of the CDSS. The order (CDSS first, conventional methods second or conventional methods first, CDSS second) was the second between factor. Figure 1 shows the different conditions and orders of our design and the resulting groups for each case and order of methods used.

We calculated the sample size required for our study using G*Power 3.1 [33] based on the following criteria: *d* = 0.50, α = 0.05, 1-β = 0.80 arriving at a required sample size of *n* = 34 per group, *N* = 136 in total. Anticipating a dropout rate of 20%, we aimed to recruit 164 participants. We used the two pilot tutorials to assess the mean difference for the within-subjects effect (CDSS vs. conventional methods).

### CDSS: Ada^®^ mobile application

As CDSS, participants used the symptom checker Ada^®^ versions 3.3.0 until 3.7.0 from Ada Health GmbH. We chose the free of charge app Ada^®^ which is easily available in respective app stores for Android- and iOS-based smartphones. Also, the question-based approach of Ada^®^ mimics to some degree the conventional medical history taking of signs and symptoms. After providing basic health information, the user enters the main complaints. Ada^®^ then asks a series of follow-up questions through a reasoning engine which bases its questions, diagnoses, and recommendations on probability estimations and its medical knowledge database [34]. For this study, students entered the information based on the assigned case vignette into the application.

### Questionnaire and assessment of diagnoses

The questionnaire contained 63 items. After providing informed consent, students were instructed to use first the CDSS (groups A and C) or, alternatively, conventional methods (groups B and D) to make a diagnosis based on the paper case. Subsequently, participants provided up to three diagnoses, each accompanied by an indication of how confident the students felt in view of their own diagnoses (0–100% for the conventional case solving or “_ of 10” to “_ of 1,000” patients based on the CDSS output). The same case description was provided a second time with the instruction to use conventional methods (groups A and C) or the CDSS (groups B and D), i.e., those methods not used in the first run. Again, participants could indicate up to three diagnoses along with the corresponding assessment of trust in their own diagnoses. When students had to use conventional methods students had to indicate which resources they frequented. Sociodemographic information concluded the online questionnaire with questions on age, sex, semester of study, and prior medical experience, as well as a question asking for further comments.

Three external consultants in general medicine, internal medicine, and otorhinolaryngology assessed accuracy of the diagnoses suggested by students. Each expert was given all diagnoses for both cases (*N* = 46 for case 1 and *N* = 98 for case 2), the case descriptions, and ten additional diagnoses randomly chosen from the ICD-10 for each case. This latter procedure ensured that the assessment of accuracy in both the typical and the atypical presentation was absolute rather than relative and similarly calibrated. Each diagnosis was assessed on a scale from 1 (incorrect) to 5 (correct) using the organ system and the disease mechanism as points of reference and was modelled after studies similar to our own [35, 36]. If both were correct, the diagnosis was given 5 points; if both were incorrect, the diagnosis was given 1 point; if one was correct, the other incorrect, the diagnosis received 3 points. The scores 2 and 4 paid heeds to grey areas common in medical practice which is why we chose to involve the three outside experts. Using the median score from the three experts identified the final scores for each diagnosis. The highest scoring diagnosis for each participant in each condition was included in the analysis.

### Confounders

We undertook the following measures to mitigate possible effects of the confounders that might have influenced our study: we employed random assignment of students to cases and order, taking into account the possibility of a selection bias as some students of our faculty may have already had prior exposure to advanced technological systems and were more technically proficient than others and were hereby more interested in the topic of the tutorial and our study. However, we believe that technical proficiency does not help providing more accurate diagnoses. Although later versions of the CDSS may have potentially yielded improved results, there were no significant differences in diagnostic accuracy observed across different versions of the CDSS. Students who participated at a later date might have had knowledge of the cases due to their interactions with students who took part in the study at an earlier date. It is important to note that we explicitly instructed all students not to discuss the study with other interested students and never disclosed any diagnoses during the tutorials. Finally, it is possible that students who were in the later stages of their medical studies had a higher level of proficiency compared to students in earlier stages. However, our findings did not indicate any significant effect.

### Analysis

We identified the percentage of missing data in the dataset and calculated Little’s missing completely at random (MCAR) test to check whether missing data were distributed randomly across variables. We used *t*-tests with independent samples for gender differences regarding all outcomes to check for potential gender impacts on our analyses. To test the primary hypothesis, we calculated a *t*-test with paired samples between the CDSS condition and the conventional condition. We then looked for effects of case (difficulty) and order (CDSS/conventional first) using a mixed analysis of variance (ANOVA). For trust in the diagnoses between cases, we calculated a *t*-test for independent samples between case 1 and case 2. For trust in the diagnoses depending on order, we calculated a *t*-test for paired samples comparing groups A and B with groups C and D. For effects of case and order on trust in the diagnoses, we then used a univariate ANOVA. We used SPSS 27.0 with its respective effect size calculations and pairwise deletion for all analyses. We used the descriptive statistics of mean and standard deviations for illustrating our results in tables and figures.

## Results

### Sample

Two hundred thirteen medical students at Charité – Universitätsmedizin Berlin participated in the study. We excluded 47 students, because they did not meet the inclusion criteria regarding their year of study, or having participated in the tutorial before, or for being affiliated with Ada Health GmbH. The remaining *N* = 166 students (57% female) aged *M* = 25.31 years (*SD* = 4.49 years) were included in the analysis. Table 1 shows the distribution among the four groups.

**Table 1:** Sample characteristics

Variable	Group A	Group B	Group C	Group D	*F/χ*^*2*^	*p*
N	43	40	39	44	-	-
Sex (% female)	58%	57%	36%	71%	11.52	.01
Age, years, mean (SD)	24.85 (3.45)	24.71 (3.24)	25.32 (4.88)	26.32 (5.78)	1.05	.37
Semester, mean (SD)	8.40 (0.98)	8.83 (1.01)	8.46 (1.17)	8.34 (1.08)	1.74	.16

Ten individuals did not report their age. *SD* Standard deviation. We report F-values for ANOVAs with age and semester, and a χ^2^-value for gender

Randomisation provided equal distribution regarding age and semester, but unequal distribution of gender in the four groups. We accordingly analysed gender differences on our outcomes. Table 2 provides results for three *t*-tests. Men and women did not differ significantly regarding their accuracy with the CDSS or with conventional methods. They also did not differ significantly in the trust in their diagnosis. We thus do not consider the unequal distribution of gender to influence the results of our study regarding accuracy and trust in the diagnoses.

**Table 2:** Gender differences regarding the outcomes accuracy and trust

Variable	**Women**	**Men**	**t**	*p*
Accuracy CDSS mean (SD); n	3.79 (1.29); 94	3.76 (1.32); 72	0.12	.91
Accuracy conventional mean (SD); n	3.95 (1.28); 93	3.82 (1.33); 72	0.62	.54
Trust conventional mean (SD); n	60.92 (21.16); 92	62.44 (22.57); 71	-0.44	.66

*SD* Standard deviation

### Missing data

The final dataset contained 3% missing data. Little’s MCAR test provided significant results (χ2 = 2408.58, df = 2221, *p* = 0.003) meaning data were not missing completely at random. Accordingly, we checked all variables with missing data for any patterns of missingness, and identified the pattern that the accuracy and trust values of the second and third CDSS diagnoses were always missing in respective pairs. Together, these variables accounted for more than 50% of missing data. We thus excluded the variables from our missing data analysis to identify patterns beyond this technical covariation. After excluding the variables, Little’s MCAR test provided non-significant results (χ2 = 1159.57, df = 1095, *p* = 0.086). Thus, we assumed data in our dataset to be missing completely at random.

### Conventional methods used

The most common sources of information in the conventional groups were websites (69%) and analogue textbooks (9%). The most visited website was *Amboss* – a German digitalised textbook-like aid used by medical students primarily to prepare for the nationwide licencing exams covering all fields of medicine (79%) followed by the German medical wiki *DocCheck Flexikon* (19%), and Wikipedia (4%). Twenty participants (12%) reported solving the case by themselves without further aids.

### Accuracy of diagnoses

**Fig. 2:**
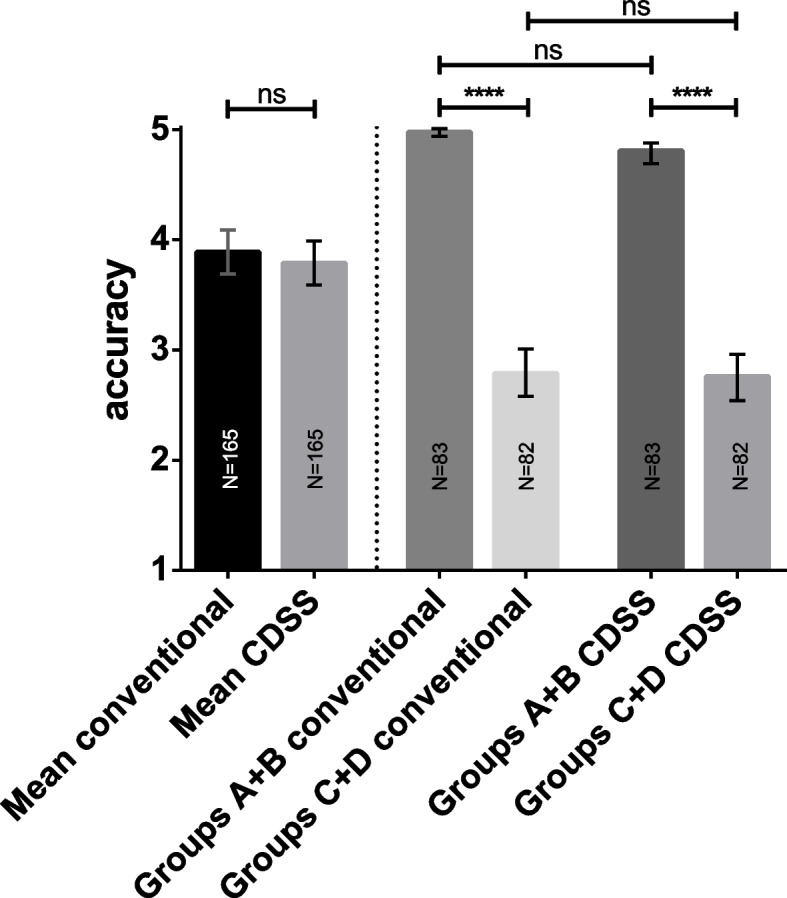
Accuracy of diagnoses. Bars show mean diagnostic accuracies for both methods (conventional and CDSS) across cases (left side) as well as within cases (right side). There is no statistically significant difference in accuracy across and within cases for conventional diagnosis in comparison with the CDSS. Students provided more accurate diagnoses in case 1 in comparison to case 2 using either method. ****, *p* < .0001 (significant), ns, non-significant. The number of individual students in each cohort is provided in each bar. Whiskers indicate standard deviations representing 68% of values around the mean

Figure 2 shows the results of our primary hypothesis concerning the diagnostic accuracy. Diagnoses for conventional resources were similar to using CDSS across and between cases. Diagnoses for case 1 were significantly more accurate than case 2 using both conventional methods and the CDSS. For case 1, 81 out of 83 students scored an accuracy of 5 and the other two students scored an accuracy of 4 points. For case 2, one student out of 82 was rewarded with a score of 5 and 29 were rewarded with a score of 4.

Table 3 shows the mixed ANOVA results for the diagnostic accuracy, the within-subjects condition conventional/CDSS, and the two between-subjects conditions case and order as independent variables. Only the between-subjects condition *case* had a significant effect on the accuracy of diagnosis.

**Table 3:** Mixed ANOVA results for accuracy of diagnoses

Predictor	Sum of squares	df	F	*p*	η
Tests of within-subjects effects					
Constant					
Method	0.78	1	1.49	.22	0.009
Method*case	0.38	1	0.74	.39	0.005
Method*order	0.09	1	0.18	.68	0.001
Method*case*order	1.26	1	2.42	.12	0.015
Error(Method)	83.92	161	-	-	-
Tests of between-subjects effects					
Case	369.01	1	639.47	< .01	0.799
Order	0.01	1	0.02	.88	0.000
Case*order	1.28	1	2.22	.14	0.014
Error	92.9	161	-	-	-

*N* = 165; method = within-subjects condition using the CDSS or conventional methods; case = between-subjects condition processing case 1 or case 2; order = between-subjects condition using the CDSS first or second; *indicates interactions between the respective variables; R^2^ = .80

Later versions 6.0 and 7.0 of the CDSS did not produce more accurate diagnoses in ANOVAs than earlier version 4.0 and 5.0 for case 1 (*F*(3,72) = 0.74, *p* = 0.53, n_4.0_ = 24, n_5.0_ = 24, n_6.0_ = 17, n_7.0_ = 11) or case 2 (*F*(3,65) = 1.66, *p* = 0.19, n_4.0_ = 9, n_5.0_ = 13, n_6.0_ = 24, n_7.0_ = 23), nor did students of higher semesters 9 or 10 perform better in ANOVAs in case 1 (*F*(3,79) = 0.47, *p* = 0.71, n_7_ = 15, n_8_ = 20, n_9_ = 31, n_10_ = 17) or case 2 (*F*(3,78) = 0.77, *p* = 0.51, n_7_ = 25, n_8_ = 13, n_9_ = 29, n_10_ = 15) than their less experienced fellow students in semester 7 or 8.

### Students’ trust in own diagnoses

**Fig. 3:**
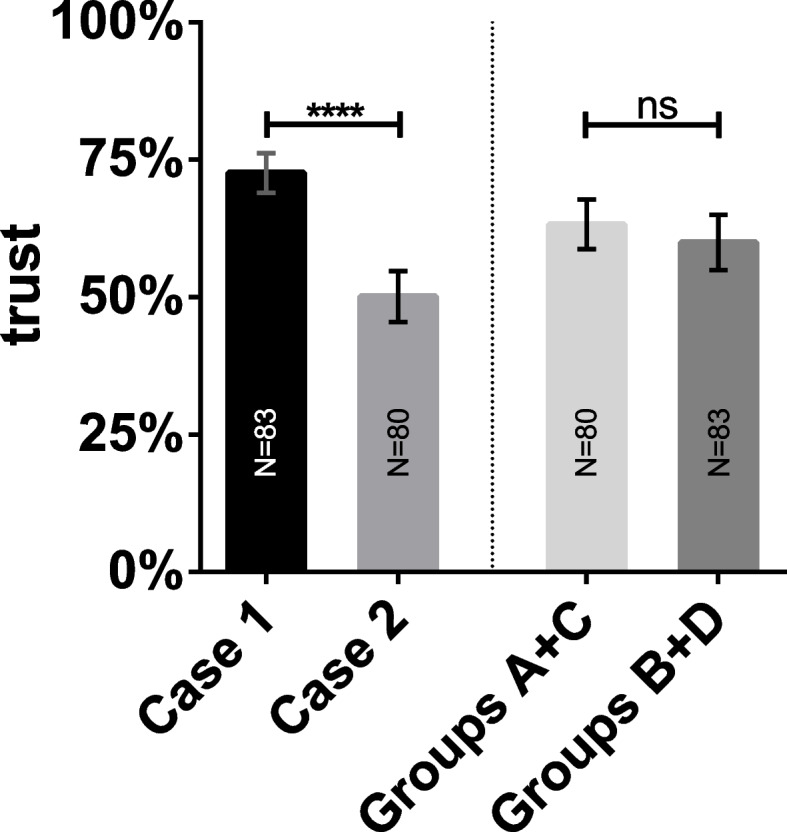
Students’ trust in own diagnoses. Bars show student’s trust in their own diagnoses (conventional condition) between cases (left side) and regarding the order of methods as between factor (right side). Students trust in their own diagnoses was significantly higher in case 1 in comparison to case 2. When comparing the order in which the diagnosis was made, no significant effect was found. ****, *p* < .0001 (significant), ns, non-significant. The number of individual students in each cohort is provided in each bar. Whiskers indicate standard deviations representing 68% of values around the mean

Figure 3 shows the results of our main and second hypothesis for the secondary outcome trust. Students’ trust in their own diagnosis was higher for case 1. No effect on trust was found concerning the order in which diagnoses were made.

**Fig. 4:**
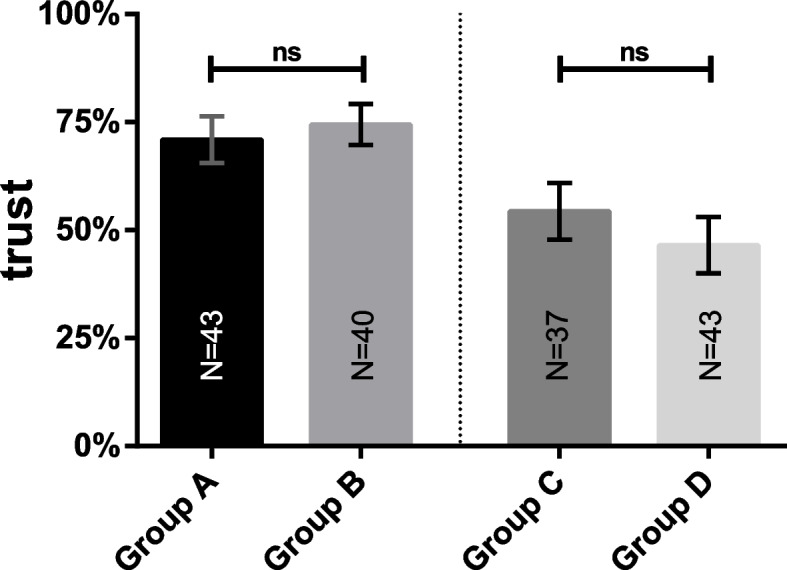
Comparison of students’ trust in own diagnoses within cases. Bars show student’s trust in their own diagnoses for case 1 (left side) and case 2 (right side). Groups differ in the order of methods (conventional vs. CDSS). There was no significant effect of method order in any case condition. ns, non-significant. The number of individual students in each cohort is provided in each bar. Whiskers indicate standard deviations representing 68% of values around the mean

Figure 4 and Table 4 highlight some interaction effects in regard to the outcome student’s trust in their own diagnosis with the two between-subjects conditions case and order as independent variables. However, the effect for both cases was not significant.

**Table 4:** ANOVA results for trust in diagnoses

Predictor	Sum of squares	df	F	p	η
Case	20,089.18	1	58.69	< .001	0.270
Order	191.59	1	0.56	.46	0.004
Case*order	1303.76	1	3.81	.05	0.023
Error	54,425.74	159	-	-	-

*N* = 163, case = between-subjects condition processing case 1 or case 2; order = between-subjects condition using the CDSS first or second; *indicates interactions between the respective variables; R^2^ = .28

Students in higher semesters did not trust their diagnoses more than students in lower semesters in ANOVAs for case 1 (*F*(3,79) = 2.59, *p* = 0.06, n_7_ = 15, n_8_ = 20, n_9_ = 31, n_10_ = 17) or case 2 (*F*(3,76) = 0.83, *p* = 0.48, n_7_ = 24, n_8_ = 13, n_9_ = 28, n_10_ = 15).

## Discussion

Our aim was to evaluate students’ diagnostic accuracy and trust in their diagnoses using conventional methods and a CDSS in two clinical scenarios with different degrees of difficulty. When a common disease presenting with typical signs and symptoms was concerned, students reached a very high level of diagnostic accuracy close to 100%. In the case of a common disease presenting in an atypical way, students’ accuracy decreased substantially. In both scenarios, we found no significant difference in accuracy when students used conventional methods or relied on the output of the CDSS. This result leads us to refute our original hypothesis that students would come to more accurate diagnoses when they use conventional methods as compared to the use of a CDSS.

Obviously, between the two cases there was a relevant difference in students' trust in their own diagnoses: in the typical case condition, students exhibited higher trust in their diagnoses while in the atypical case trust was much lower. But, in sharp contrast to the very high accuracy with which they solved the typical case students had much less trust in their own diagnosis. Conversely, for the case with the atypical presentation, average accuracy ratings paralleled average trust ratings. In addition, we also observed another slight, though not statistically significant, influence on the students’ trust in their diagnoses: the latter was lower when the CDSS was used first for the typical case and higher when they used the CDSS first in the atypical case.

Our findings suggest that students' diagnostic accuracy in common diseases is strongly influenced by the presence of typical signs and symptoms and less channelled by technological support. The accuracy observed in the typical case indicates that students have the ability to correctly diagnose a textbook-like presentation with which they are familiar. In contrast, their diagnostic performance declines when confronted with an atypical presentation, even of a common disease. This observation highlights the challenges students face in recognising uncommon manifestations of common diseases.

We can show that the CDSS did not provide a particular help to improve students' ability to come to accurate diagnostic conclusions. However, the CDSS may play a notable role in enhancing students' trust and confidence especially when students are confronted with more complex cases. In these scenarios the CDSS may act almost like a second opinion and as a source of reassurance which gives students greater confidence in their own diagnostic decision-making process. In this sense the use of the CDSS may offer valuable psychological support as a tool to boost students' confidence and decision-making skills. Therefore, it would be helpful in the future to explore more the psychological and educational impacts of CDSS in different diagnostic scenarios and with larger sample sizes. Additionally, our study provides in an exemplary fashion how an experimental set-up could work to assess other dimensions of student`s diagnostic accuracy, i.e. rare diseases or diagnoses in morphological fields such as radiology or pathology.

Hitherto, the focus of the research revolving around supporting devices for medical decision-making has almost exclusively addressed rare diseases or challenging and unusual clinical constellations [21]. Because of this rather narrow perspective, we chose common diseases with different presentations to better reflect the students' level of knowledge and their educational contexts as students had to come to their own diagnostic conclusions rather than to select one diagnosis from a set of prefabricated diagnoses as it is the case e.g. in multiple-choice questions commonly used in medical education [37]. We believe that our approach allowed for a more comprehensive investigation of the impact of CDSSs in the context of more commonly encountered clinical scenarios in medical education as students are only in the process to learn how to classify, assess, weight, interpret and finally synthesise subjective complaints and objective clinical signs as Bowen showed [5].

Also, we consider it to be a particular strength of our study that we did not measure diagnostic accuracy in a dichotomous way that only differentiates between correct or incorrect. To refine our results we developed a scale ranging from 1 to 5 based on Ramnarayan et al. [35] and Bond et al. [36] which takes into account parameters such as affected organ systems and disease mechanisms. Clinical practise generally implies many more nuances between an entirely correct and an entirely incorrect diagnosis. Also, a graded spectrum of partially correct answers reflects much better the various aspects that students need to synthesise in their diagnostic reasoning. As such the path to become a diagnostician is much better reflected by the scale we used than by an exclusively correct – incorrect alternative. Obviously, for other research purposes such a scale can be modified in different ways according to the eventual research questions at stake. Such a scale may also be used as a tool to grade potential progress in the learning curve of an individual. Without doubt, further studies are warranted to also test the validity of our approach.

### Limitations

A major methodological limitation of our study relates to the fact that students were simply instructed not to use the CDSS when they were asked to use conventional methods. As a result, the majority of students used a variety of online services such as *Amboss* or *DocCheck Flexikon*, and only a minority went back to hard-copy textbooks and guidelines, other students did not look for any additional help at all. Thus, the conventional condition consists of a broader and much more heterogeneous range of mostly digitalised resources medical students are familiar with altogether. Therefore, students might have retrieved very different information to come to a diagnostic conclusion whereas the use of the CDSS represents a uniform pathway.

## Conclusion

To summarise, the use of a CDSS did not significantly improve diagnostic accuracy. Nevertheless, the use of such a software may play an interesting and also relevant role in enhancing students' trust and confidence when presented with challenging clinical constellations. We also observed that students tend to underestimate their own knowledge in easy, typical cases, while they are much more aware of their limitations in challenging, atypical cases. Our results also highlight the significance to consider both objective as well as subjective parameters when evaluating the effectiveness of CDSS. Using CDSS in medical education, instructors could eventually optimise the integration of this technology in the training of future physicians.

## Data Availability

The dataset supporting the conclusions of this article is available in the Open Science Framework, [https://osf.io/aegcp/files/osfstorage].

## Abbreviations

CDSS: Clinical decision support system
Little’s MCAR test: Little’s missing completely at random test
ANOVA: Analysis of variance
